# Listeria monocytogenes Spontaneous Bacterial Peritonitis in a Patient With Decompensated Cirrhosis

**DOI:** 10.7759/cureus.100155

**Published:** 2025-12-26

**Authors:** Leen M Ahmed, Sara M Farah, Layan Alali, Majd Hamzeh, Ahmed M Saleh

**Affiliations:** 1 Faculty of Medicine, Hashemite University, Zarqa, JOR; 2 Internal Medicine, Henry Ford Rochester Hospital, Rochester Hills, USA; 3 Internal Medicine, Wayne State University School of Medicine, Detroit, USA

**Keywords:** alcoholic cirrhosis, ampicillin, ascites, listeria monocytogenes, listeria sbp, spontaneous bacterial peritonitis (sbp)

## Abstract

A 54-year-old man with decompensated alcohol-related cirrhosis presented with worsening abdominal pain, recurrent ascites, and acute kidney injury on chronic kidney disease. Initial diagnostic paracentesis showed a polymorphonuclear (PMN) count of 75 cells/μL, which lowered suspicion for spontaneous bacterial peritonitis (SBP). However, ascitic fluid culture subsequently isolated *Listeria monocytogenes*, a rare cause of SBP that is intrinsically resistant to third-generation cephalosporins. Empiric ceftriaxone would not provide reliable coverage. Therapy was escalated to high-dose intravenous ampicillin, with subsequent transition to oral trimethoprim-sulfamethoxazole to complete therapy, together with large-volume paracentesis, albumin supplementation, and supportive management of hepatorenal dysfunction. The patient improved clinically and biochemically and was discharged in stable condition. This case underscores the need to consider *Listeria* in cirrhotic patients who fail to respond to standard cephalosporin-based therapy and to promptly tailor antibiotics to culture results to optimize outcomes.

## Introduction

Spontaneous bacterial peritonitis (SBP) is a serious complication of cirrhosis, defined as infection of ascitic fluid without an intra-abdominal surgical source [1]. It affects approximately 10% of hospitalized patients with cirrhosis and carries high morbidity and mortality [1,2]. SBP is most commonly diagnosed when ascitic fluid analysis demonstrates a polymorphonuclear (PMN) cell count ≥250 cells/µL in the absence of an alternative intra-abdominal source of infection; however, culture-positive infections with lower PMN counts have been described, particularly in early infection or with atypical organisms. The majority of SBP cases are caused by enteric Gram-negative organisms, including *Escherichia coli* and *Klebsiella pneumoniae*, although Gram-positive cocci such as *Streptococcus pneumoniae* can also be involved [2].

*Listeria monocytogenes* is an uncommon cause of SBP, more typically associated with meningitis and bacteremia in immunocompromised hosts [3]. *Listeria *SBP remains exceptionally rare, with only isolated case reports and small case series reported in the literature [3,4]. This organism is clinically significant because *Listeria *species are intrinsically resistant to third-generation cephalosporins, the standard first-line therapy for SBP [4,5]. Delayed recognition and inappropriate empiric therapy can contribute to adverse outcomes.

We present this case to highlight the diagnostic challenges posed by *L. monocytogenes *as a rare cause of SBP, particularly in the setting of low ascitic fluid PMN cell counts and intrinsic resistance to standard empiric therapy. This report underscores the importance of early microbiological evaluation and maintaining a broad diagnostic approach in patients with decompensated cirrhosis who fail to respond to conventional treatment.

## Case presentation

In July 2025, a 54-year-old man with advanced decompensated alcohol-related cirrhosis, prior hepatic encephalopathy, esophageal varices, and chronic kidney disease presented with severe abdominal pain, progressive abdominal distension, and generalized swelling of the face and limbs consistent with anasarca. He had recent admissions for ascites and presumed hepatorenal syndrome. On arrival, his temperature was 98.1°F, with blood pressure of 115/69 mmHg, heart rate of 120 beats/min, respiratory status being stable with oxygen saturation 98% on room air, and without overt encephalopathy. The abdomen was distended with diffuse tenderness. Initial laboratory results are summarized in Table 1.

**Table 1:** Initial laboratory results Initial laboratory values obtained at the time of presentation, including electrolyte, renal, hepatic, and hematologic parameters.

Parameters	Result	Unit	Reference Range
Sodium	128	mEq/L	135–145
Blood urea nitrogen	42	mg/dL	7–20
Creatinine	2.36	mg/dL	0.6–1.2
Total bilirubin	1.4	mg/dL	0.1–1.2
Alkaline phosphatase	267	U/L	44–147
Aspartate aminotransferase (AST)	122	U/L	10–40
Lipase	252	U/L	0–160
Serum albumin	1.3	g/dL	3.5–5.0
Hemoglobin	10.6	g/dL	12.0–16.0 (female), 13.5–17.5 (male)
Red cell distribution width (RDW)	17.8	%	11.5–14.5
White blood cell count (WBC)	22.4	×10^3^/µL	4.0–11.0

Diagnostic paracentesis yielded clear yellow fluid with a total white blood cell count of 150/µL and polymorphonuclear cells 75/µL. Given his clinical deterioration and concern for spontaneous bacterial peritonitis, empiric intravenous ceftriaxone was initiated while awaiting culture results. Ascitic fluid culture subsequently grew *L. monocytogenes*, prompting a switch to high-dose intravenous ampicillin during hospitalization. He underwent repeat large-volume paracentesis for symptom relief during the admission. Renal dysfunction was managed with intravenous albumin supplementation alone, without the use of vasoconstrictor therapy.

Renal function improved to baseline, and abdominal pain resolved. He was discharged after a five-day hospitalization with instructions to complete the antibiotic course. He received IV ampicillin until discharge and was discharged on oral trimethoprim-sulfamethoxazole (TMP-SMX) for an additional 14 days to complete therapy, with outpatient follow-up arranged. A timeline of the patient’s clinical course is shown in Table 2.

**Table 2:** Timeline of the clinical course The patient presented with abdominal pain and tense ascites and underwent diagnostic paracentesis with 8.3 L removed. Empiric ceftriaxone therapy was initiated. Ascitic fluid culture returned positive for *Listeria monocytogenes* on hospital day two, prompting a switch to intravenous ampicillin. A repeat paracentesis with 4 L removed was performed on day three for symptom relief. The patient demonstrated improvement in abdominal pain and renal function and was discharged on day five to complete antibiotic therapy with oral trimethoprim-sulfamethoxazole and scheduled outpatient follow-up.

Day	Clinical Events
Day 0	Presented with abdominal pain and tense ascites
	Diagnostic paracentesis performed (8.3 L removed)
	Ascitic WBC 150/µL, PMN 75/µL
	Started IV ceftriaxone
Day 2	Ascitic fluid culture positive for Listeria monocytogenes
	Switched to IV ampicillin
Day 3	Repeat paracentesis performed (4 L removed)
	Clinical improvement noted
Day 5	Renal function returned to baseline
	Abdominal pain resolved
	Discharged to complete antibiotic therapy with oral trimethoprim-sulfamethoxazole and scheduled for outpatient follow-up

The patient remained clinically stable following discharge. He was prescribed oral TMP-SMX to complete a planned 14-day course; however, he continued taking the medication for approximately 30 days based on available supply. Outpatient clinic follow-up was scheduled 10 days after discharge, but further follow-up was limited due to insurance constraints and transportation difficulties. There has been no documented recurrence of *L. monocytogenes* infection since discharge. The patient continues to undergo intermittent therapeutic paracentesis for management of ascites related to cirrhosis.

## Discussion

*L. monocytogenes *is a rare but clinically important cause of spontaneous bacterial peritonitis (SBP), most commonly reported in patients with advanced cirrhosis or impaired host defenses [3,4]. Although SBP is typically caused by enteric Gram-negative organisms such as *E. coli *and *K. pneumoniae*, *L. monocytogenes *represents an important atypical pathogen that should be considered in selected clinical contexts [2-4].

Unlike typical SBP pathogens, *Listeria *species are intrinsically resistant to third-generation cephalosporins, which remain the standard empiric therapy for SBP in many clinical settings [1,2,6]. Consequently, failure to recognize *L. monocytogenes *as the causative organism may delay initiation of appropriate antimicrobial therapy and adversely affect outcomes [3-5]. Reviews of listeriosis management consistently identify ampicillin-based regimens as the treatment of choice for invasive *Listeria *infections [5].

SBP is conventionally diagnosed when the ascitic fluid polymorphonuclear (PMN) cell count is ≥250 cells/µL in the absence of an alternative intra-abdominal source of infection [1,2]. Most reported cases of *Listeria*-associated SBP describe PMN counts exceeding this diagnostic threshold [3,4]. In contrast, our patient demonstrated culture-positive ascitic fluid with a PMN count below 250 cells/µL, consistent with monomicrobial non-neutrocytic bacterascites, a recognized but less common variant of ascitic fluid infection [7].

Several mechanisms may contribute to low ascitic neutrophil counts in this setting, including early infection, cirrhosis-associated immune dysfunction, and impaired neutrophil recruitment in advanced liver disease [7,8]. This variability in inflammatory response highlights the importance of obtaining ascitic fluid cultures even when PMN counts do not meet conventional diagnostic criteria, particularly in high-risk cirrhotic patients with systemic signs of infection [2,7].

Recognition of *Listeria *SBP is particularly important given its intrinsic resistance to standard empiric cephalosporin therapy. Prior case reports have described delayed clinical improvement until antimicrobial therapy was modified to include ampicillin-based regimens [3,4,6,9]. Early microbiological evaluation and prompt culture-directed adjustment of antibiotics therefore remain essential components of effective management.

Supportive management in SBP includes close monitoring of renal function, especially in patients with advanced cirrhosis who are at increased risk for renal impairment [1]. Therapeutic paracentesis may be required for symptomatic relief of ascites in selected cases [1].

This case reinforces the importance of maintaining a high index of suspicion for atypical organisms such as *L. monocytogenes *in cirrhotic patients who fail to improve on standard empiric SBP therapy. It also highlights that culture-positive SBP may occur even when ascitic PMN counts are below the traditional diagnostic threshold, underscoring the critical role of microbiological testing in guiding appropriate therapy [7,9].

## Conclusions

This case emphasizes the importance of obtaining early ascitic cultures and reassessing treatment response in patients with spontaneous bacterial peritonitis. The lack of improvement with standard cephalosporin therapy should prompt consideration of atypical pathogens such as *L. monocytogenes *and initiation of appropriate antibiotics. Timely culture-guided therapy remains essential to optimize outcomes in vulnerable cirrhotic patients.
